# Retention of implant retained obturator using two implant placement configurations for maxillectomy cases: in-vitro study

**DOI:** 10.1186/s12903-024-04797-3

**Published:** 2024-09-11

**Authors:** Nourhan I. Aboseada, Faten S. Mohamed, Sonia M. El-shabrawy

**Affiliations:** 1 Faculty of Dentistry, Alexandria University, Alexandria, Egypt; 2 Prosthodontics Department, Faculty of Dentistry, University of Alexandria, Alexandria, Egypt; 3 Biomaterials Department, Faculty of Dentistry, University of Alexandria, Alexandria, Egypt

**Keywords:** Implant-retained obturator, Implant configuration, Ball and socket, Maxillectomy

## Abstract

**Background:**

Implant-retained obturators for maxillectomy cases have several advantages over traditional obturators but prosthetic design for specific conditions after maxillary resection has several challenges and the appropriate implant placement configuration is essential for improving retention and the stability of the implant-retained obturator.

**Objectives:**

The present study aimed to assess the retention force of using linear and nonlinear implant placement configurations using ball and socket attachment in implant-retained obturators at the initial retention and after simulation of six months of use.

**Materials and methods:**

Two identical epoxy resin maxillary models of a completely edentulous unilateral maxillary defect (Brown’s class IIb) were used for implant placement, in the first model three implants were arranged with linear placement configuration, and in the second model three implants were arranged in nonlinear placement configuration. For proper sample sizing, 26 models and obturator were used. Two equal groups of obturators (13 for each group) were constructed, each with a different implant placement configuration. Both groups used the same attachment design (a non-splinted ball attachment). Using a cyclic loading machine that served as a dental insertion and removal simulator, each study group was subjected to 500 tension-compression cycles simulating 6 months of use. Using the universal testing machine, each obturator was removed at a speed of 50 mm/min for the crosshead. peak load to dislodgement was measured at the initial retention and after the simulations of six months of use. Data were analyzed using independent and paired t-tests while percent change was analyzed using the Mann Whitney U test.

**Results:**

There were a statistically significant differences in retention between the nonlinear implant placement configuration for Brown’s class IIb maxillectomy and the linear implant placement configuration at initial retention evaluation with *p*-value of < 0.0001 and after simulation of six months of usage with *p*-value of < 0.0001 Also, after simulation of 6 months of use group I lose − 24.87 (10.16) % of its retention while group II lose − 17.49 (7.78) %.

**Conclusions:**

Non-linear implant placement is more retentive at the initial retention and after simulation of six months of use than linear and loses less retention after usage.

## Background

Surgical management and Prosthetic reconstruction (rehabilitation) for patients suffering from maxillary defects due to different oral malignancies are challenging. Various treatment modalities have been described in the literature based on the consistency, volume, and position of the defect and other patient factors that can influence the management whether surgical, prosthodontic, or combined rehabilitation [1, 2].

The use of surgical flaps or prosthetic obturators is considered one of the functional managements for obturating the different sizes of maxillary defects that might vary in size from small to medium, and massive defects depending on the extent of the resection [3].

The use of conventional prosthetic obturators for a completely edentulous partial maxillectomy is dependent upon several factors, including the extent of the defect, the availability of tissue undercuts surrounding the cavity, the development of muscle control, and the direct and indirect retention provided by the remaining teeth [4] which might be unsuccessful in maintaining adequate retention and support to improve oral esthetic as well as function. Therefore, the use of intraoral implants would significantly improve retention. However, maintaining osseointegration between the bone and implants through bone remodeling necessitates that the various stresses produced around the bone by the occlusal load delivered to the implant be within an appropriate range [5, 6]. Furthermore, it has been observed that overloading, through concentrating stress at the implant-bone contact, can increase bone resorption [7].

However, a lack of standard data regarding the number, distribution, length, and diameter of implants in the residual tissues following resection of the defect in maxillectomy patients is debatable, depending on the extent of the defect, the height of the residual alveolar ridge, and the amount and shape of the remaining palatal shelf considered variable among different defect sizes [8]. Puryer J, Forbes-Haley C [9] emphasize the importance of meticulous planning for implant placement and the necessity of planning the obturator prostheses before implant surgery.

According to Roumanas et al. [10], for implant-retained obturators, four implants have been proposed in the intact site, while in the defective site, implants implanted inside surgical defects have a low chance of surviving and are challenging to recover and maintain.

For most patients who had a posterior partial maxillectomy, implants being placed in the residual premaxillary segment is regarded as the best area due to having sufficient bone volume and density as well as being located across from the area of the defect that is the most persistent along the posterior lateral wall [11]. Moreover, the occlusal load distribution was reported to be influenced by the implant’s location [12].

Besides, in terms of biomechanics, the posterior portions of an edentulous maxilla, where the main functional occlusal stress occurs during chewing, are an ideal location for endosseous implants. However, maxillary sinus pneumatization and inadequate bone volume in the posterior maxilla may prevent implant insertion [13].

Regarding the anchoring mechanism, stud attachments, O-rings, and ball attachments provide the optimum retention and stability for implant overdenture prostheses. Ball attachments are considered one of the most popular stud attachments due to their simplicity, affordability, ease of usage, and minimal chairside time requirements [14].

Since the literature lacks conclusive data about the number and configuration of implants necessary for the success of implant-retained obturators, The present study aimed to assess the retention force of using linear and nonlinear implant placement configuration using ball and socket attachment in implant-retained obturators at the initial retention and after simulation of six months of use. Therefore, the null hypothesis was that there was no significant difference in retention between obturators retained by three implants arranged in linear and nonlinear implant placement configurations.

## Materials and methods

This is an in vitro study. The sample size was calculated to be 26 specimens allocated equally between the two study groups. Group I included 13 obturator specimens fabricated and retained by linear implant placement configuration (canine, premolar, and molar areas), Group II included 13 obturator specimens retained by nonlinear configuration (central, premolar, and molar areas).

### Sample size

Sample size was estimated based on 95% confidence level to detect differences in retention between different implant configurations. El-Amier et al. [15] reported a mean (SD) difference in retention = 273.8 (155.13) between two different implant locations. The calculated 95% confidence interval = 168.72, 378.88. The minimum sample size was calculated to be 12 samples per group, increased to 13 to make up for laboratory processing errors. The total sample size = number of groups × number per group = 2 × 13 = 26 samples. MedCalc Statistical Software version 19.0.5 (MedCalc Software bvba, Ostend, Belgium; https://www.medcalc.org; 2019).

### Procedures

**Fig. 1:**
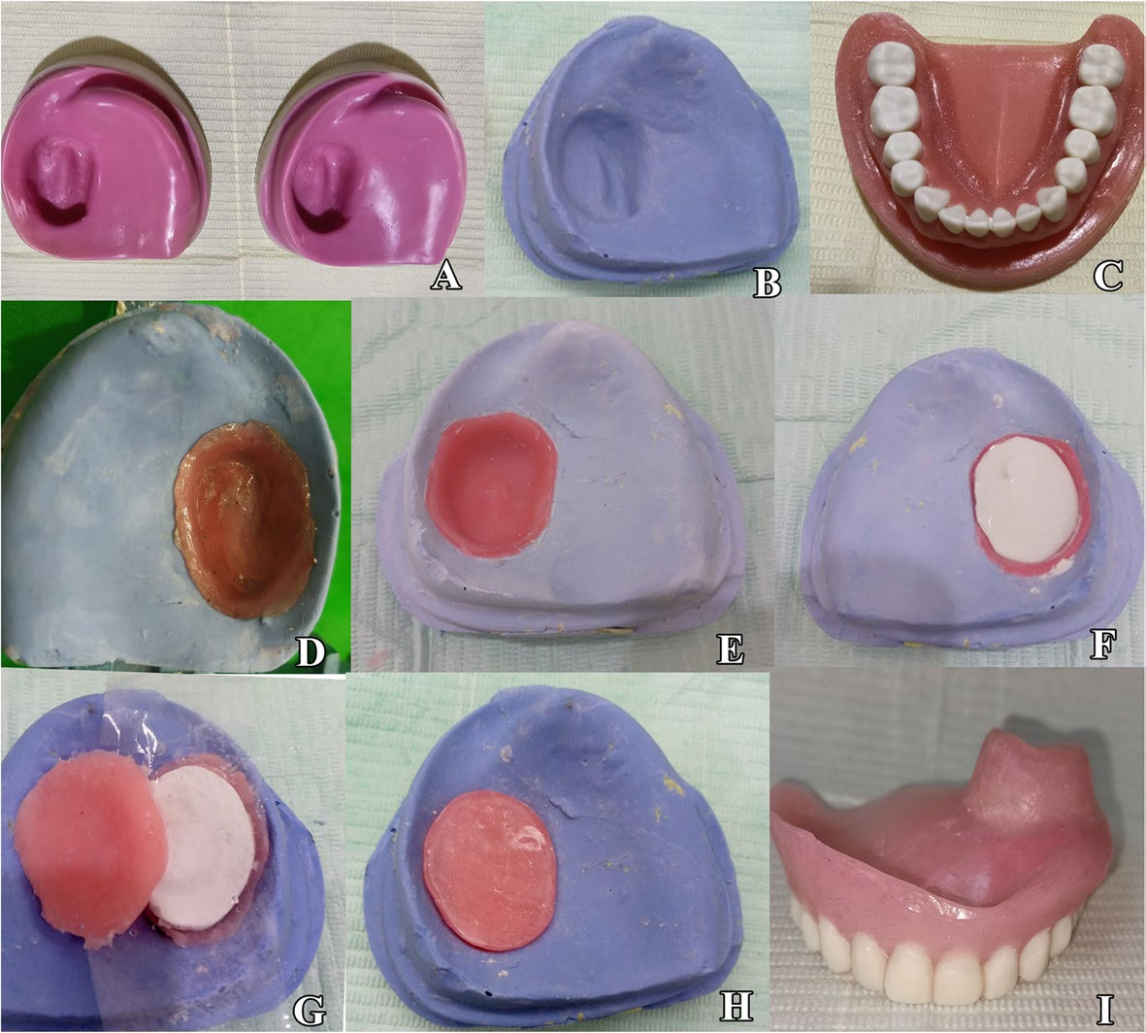
**A **Maxillary epoxy model with maxillectomy class IIb according to Brown’s classification. **B **duplicated stone casts. **C **dental Typodont Standard Teeth mandibular model** D** Two layers of base plate wax were adapted to the defect part. **E **Heat polymerized acrylic resin (Acrostone heatcure material, Cairo, Egypt) was loaded into the area and processed.** F **The interior part of the obturator was filled with soft plaster. **G **A moist cellophane paper was adapted to the obturator part's edges (**H**).The lid and the obturator component were then put together and adjusted to fit the cast (**I**).Finished obturator

Two identical standard epoxy resin completely edentulous maxillary study models having a class IIb maxillectomy arch according to Brown’s classification (Fig. 1A) [16] with mucosa stimulating material made of flexible polyurethane of 1.5 mm thickness (Ramses Medical Products Factory, Alexandria, Egypt) were used, and the study models were duplicated into twenty-six maxillary stone casts (thirteen for each group) (Fig. 1B). These casts were opposed with a fully dentate Typodont Standard Teeth mandibular model (Fig. 1C). and were used for the fabrication of closed hollow-bulb overdenture obturators.

#### Fabrication of the overdenture obturators [17, 18]

The trial obturator base with wax occlusion rim was constructed on one set of the duplicated stone models, which opposed the fully dentate Typodont Standard Teeth mandibular model and were mounted on a mean value articulator. Maxillary acrylic teeth were arranged and carefully adjusted. Twenty-six duplicated maxillary stone models were used to build 26 trial obturator bases.

To achieve standardization of all trial obturators, the opposite completely dentate Typodont Standard Teeth mandibular model with the same mounting was used to arrange the same size maxillary acrylic teeth (Acrostone cross-linked acrylic teeth, Cairo, Egypt) on all the trial obturator bases.

The obturator part was made following Elshimy’s [17] modifications to the conventional closed hollow-bulb technique as in the following procedures:

Two layers of base plate wax were applied to each cast, fitting the defect walls up to the palate edges (Fig. 1D), then flasked and rinsed away to create space for the obturator part. Heat-polymerized acrylic resin (Acrostone heat cure material, Cairo, Egypt) was loaded into the area and processed under the manufacturer’s instructions (Fig. 1E).

Lateral defect walls were sectioned to assist in retrieving the obturator part without harming the cast after cautious deflasking to preserve the cast. Without covering the margins of the obturator part, the internal space of the obturator part was filled with a lump of soft plaster and shaped to adopt the shape of the normal palatal contour (Fig. 1F).

The cover for the obturator part was then created using two layers of base plate wax. To make space for the cover component, the waxed portion was flasked and cleaned out. A moist cellophane paper was adapted to the obturator part’s edges (Fig. 1G). Heat-cured acrylic resin was packed into the space and then cured under the manufacturer’s specifications.

The lid and the obturator component were then put together and adjusted to fit the cast (Fig. 1H). After trimming the acrylic extension into the surgical defect of the trial obturator base and leaving the oral part with the waxed-up artificial teeth already placed, the waxed-up obturator base was adjusted to the cast.

The twenty-six obturator trial bases were flasked and packed using heat-polymerized acrylic resin material (Acrostone heat-cure material, Cairo, Egypt), then dentures were finished and polished (Fig. 1I).

#### Implant installation [19]

**Fig. 2:**
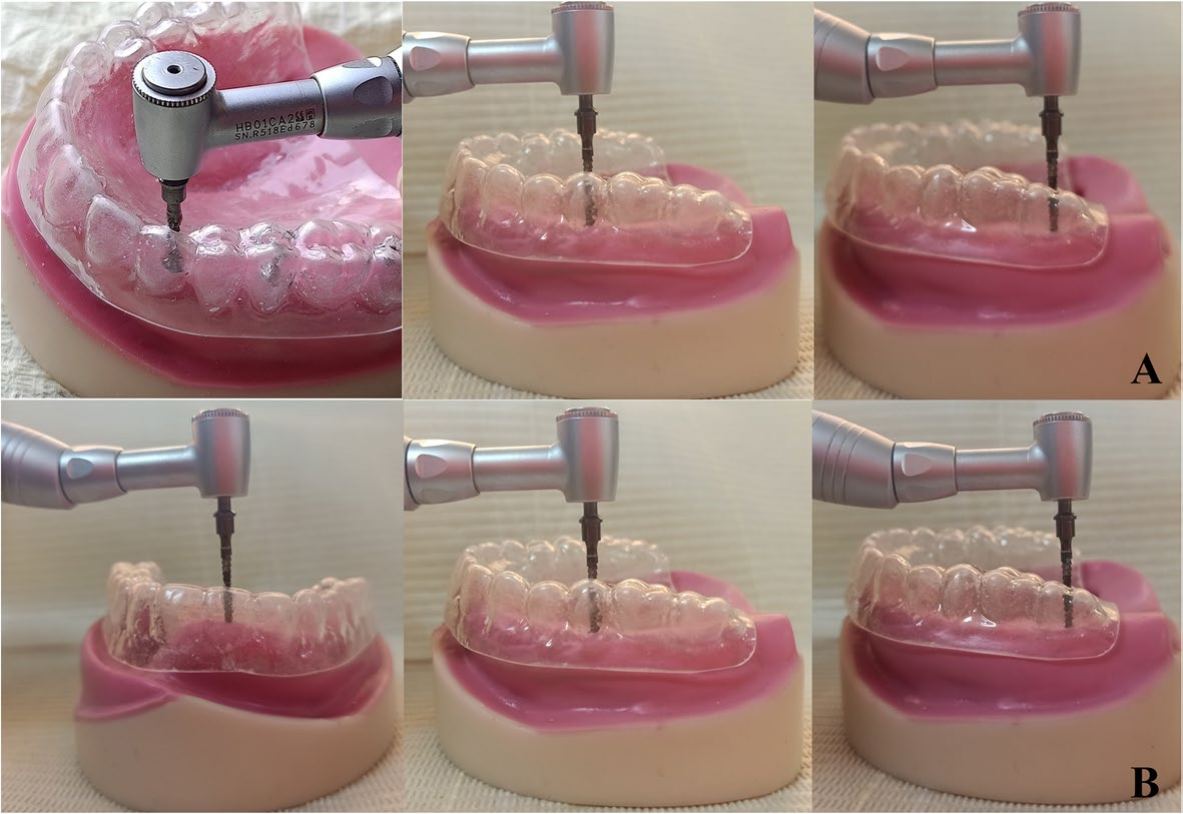
**A** Drilling of implants of model I through the acrylic templet.** B **Drilling of implant of model II through the acrylic templet

Three dummy dental implants (3.5 mm width and 10 mm length) (Dentium Superline, Dentium Co. Ltd., Korea) were placed in each epoxy study model. An acrylic drilling template (surgical template) was constructed on the duplicate of the obturator and was used for drilling at the precise implant location at the canine, premolar, and molar areas on model I, representing group I (Fig. 2A), while on model II, representing group II, the implants were drilled at the central, premolar, and molar locations (Fig. 2B).

Cortical drilling was followed by pilot drilling, body drilling (core drilling), head drilling, and then body drilling once again to clear up the debris. With the aid of a dental surveyor, and using a paralleling pin the implants’ parallelism was verified. Using a torque wrench with 35 N main stability, three implants (Dentium, Dentium Co. Ltd., Korea), each measuring 10 mm in length and 3.5 mm in diameter, were placed in the pre-drilled holes. Proper sequence and depth of drilling for implants were followed to ensure appropriate placement and stability.

#### Pick-up of ball attachment [20]

**Fig. 3:**
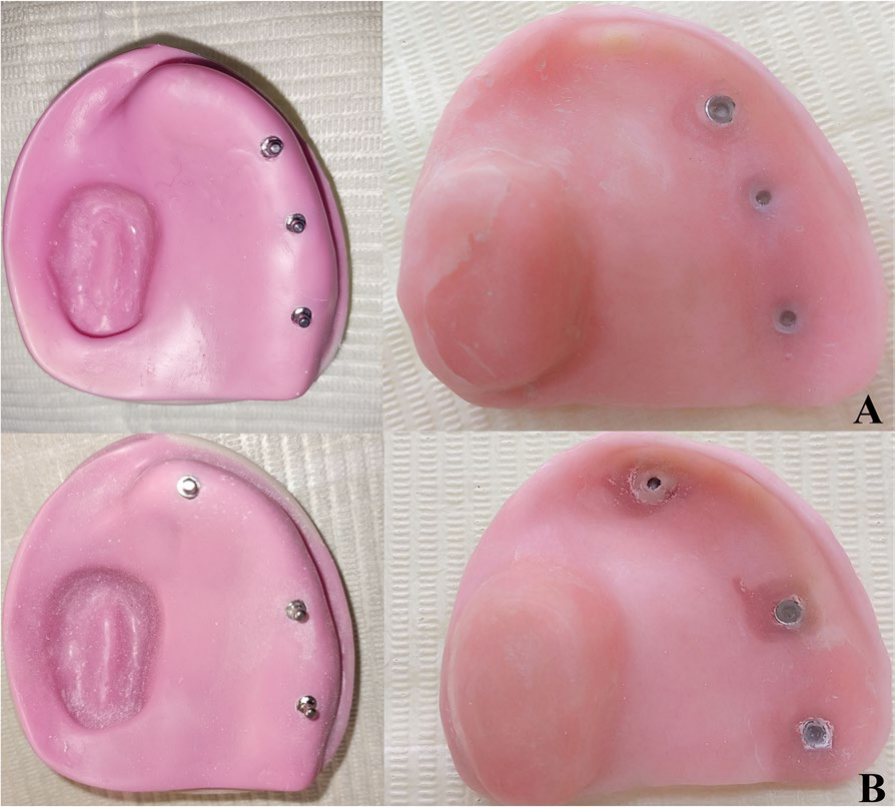
**A** Ball abutments (Rhein 83Srl, Bologna, Italy) were screwed to each implant under a torque of 20 N using a torque wrench and then the caps were picked up in the fitting surface of the obturator for group I (linear configuration) (**B**). Ball abutments (Rhein 83Srl, Bologna, Italy) were screwed to each implant under a torque of 20 N using a torque wrench and then the caps were picked up in the fitting surface of the obturator for group II (nonlinear configuration)

Ball abutments (Rhein 83Srl, Bologna, Italy) were screwed to each implant under a torque of 20 N using a torque wrench (Fig. 3A, B).

The attachments were then covered with their corresponding caps. The overdenture obturator was positioned over the maxillary model. The attachments’ locations were noted so they could be released once the obturator was fully placed. At the attachment points, three holes were drilled into the obturator’s surface to let extra self-cure acrylic resin used for attachment cap pick-up escape. On the model, a separating medium (Acrostone separating medium, Cairo, Egypt) was used, and a monomer was used on the relieved area.

A mixture of cold cure Polymethylmethacrylate was mixed. When the mixture had reached the dough stage, it was poured onto the fitting surface of the obturator. The obturator was seated above the model to pick up the caps of the attachments. The caps were initially positioned on the fitting surface of the obturator. The acrylic resin was finished and polished (Fig. 3A, B). The twenty-six obturators underwent the same process.

#### Retention evaluation

The maximum values of retention force were recorded at the beginning of the study (initial retention) and after 6 months of use, with an average of 500 cycles per 6 months based upon the patients’ average of 3 insertions and removals per day.

A universal testing machine (Mecmesin, Multi Test5-XT (5KN), USA, at the dental biomaterial department of Alexandria University) was used to dislodge the obturator. The model was fastened to the lower member of the universal testing machine.

**Fig. 4:**
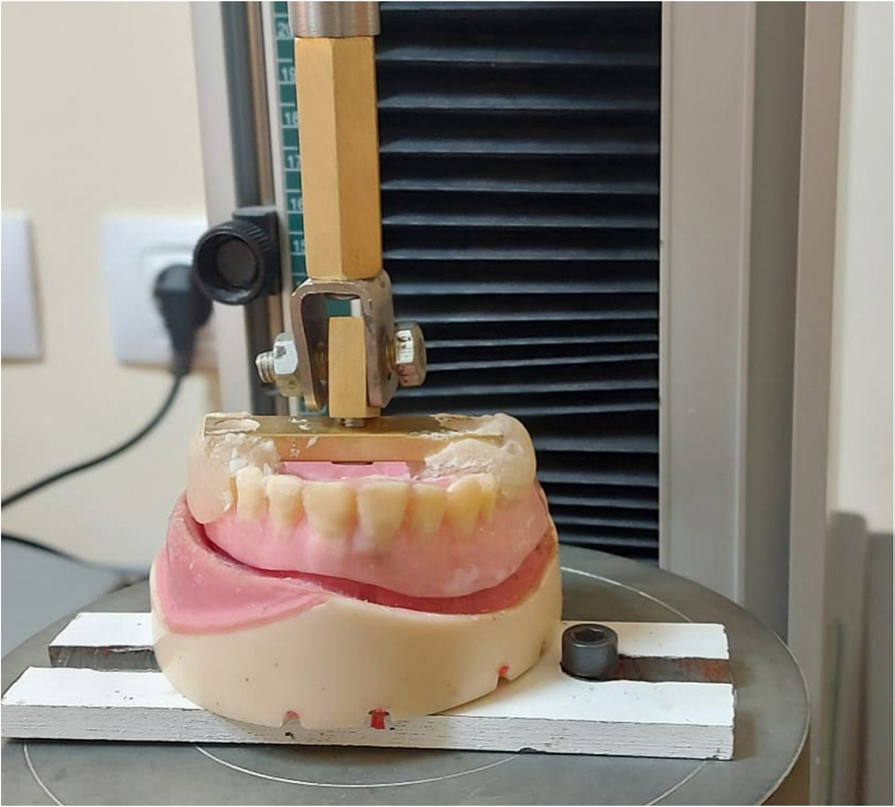
T-Shaped plate attached to the occlusal surfaces of the acrylic resin teeth at the premolars and first molar regions by using autopolymerizing acrylic resin and UTM provide a tensile dislodging force to the obturator

To provide a tensile dislodging force to the obturator, a T-shaped metal plate was attached to the upper member of the UTM (Fig. 4). The direction of the pull forces was performed vertically [21].

Auto-polymerizing acrylic resin (Special Tray Material; Acrostone Co. Ltd.) was used to attach the plate to the occlusal surface of the AR teeth in the premolar and first molar regions. Then, a tensile force was applied perpendicularly to the occlusal plane as much as possible to simulate the obturator’s axially directed dislodging forces (Fig. 4).

To withdraw the obturator, the crosshead speed of the UTM was adjusted to 50 mm/min to stimulate the dislodging speed of a prosthesis from the residual alveolar ridge during function [22] and up to an extension of 4 mm (Fig. 4).

**Fig. 5:**
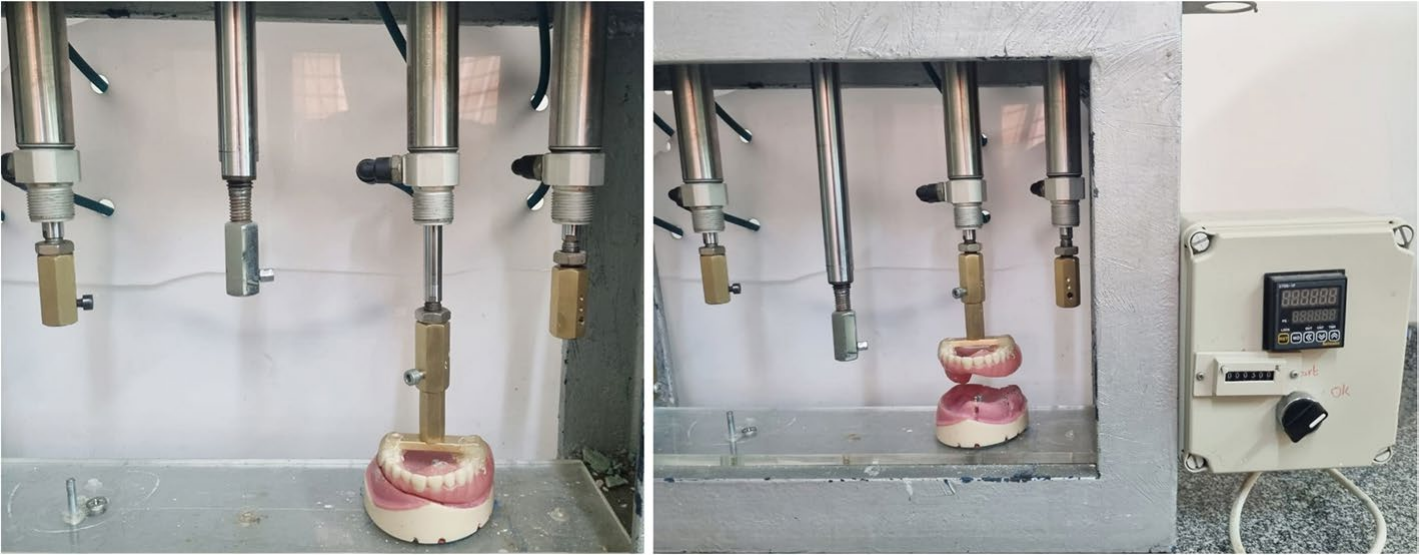
Each obturator was subjected to 500 tension-compression cycles using cyclic loading machine (insertion and removal simulator) resembling the average number of insertion and removal cycles in 6 months

A custom-made cyclic loading machine that acted as an insertion and removal simulator to simulate the insertion and removal of the 26 obturators was used to perform a cyclic tension-compression test in a vertical direction. Each obturator was subjected to 500 cycles, resembling the average number of insertion and removal cycles in 6 months based on an average of 3 removal-insertion cycles per day (Fig. 5) [23, 24].

The final retentive force of each implant placement configuration was determined by recording the peak load at dislodgement.

The resulting difference between the initial dislodgement force before cyclic loading and the final dislodgement force after cyclic loading indicated a reduction in retention by usage.

### Statistical methodology

The normality of retention values was confirmed using the Shapiro Wilk test and Q-Q plots. Percent change in retention was calculated using the following formula: [(values before treatment – values after treatment) / values before treatment] x 100. Differences in retention values were analyzed using independent and paired t-tests while percent change was analyzed using the Mann Whitney U test. All tests were two-tailed. The significance level was set at *p* value ≤ 0.05. Data were analyzed using IBM SPSS version 23, Armonk, NY, USA.

## Results

### Analysis of results for obturator retention evaluation

**Fig. 6:**
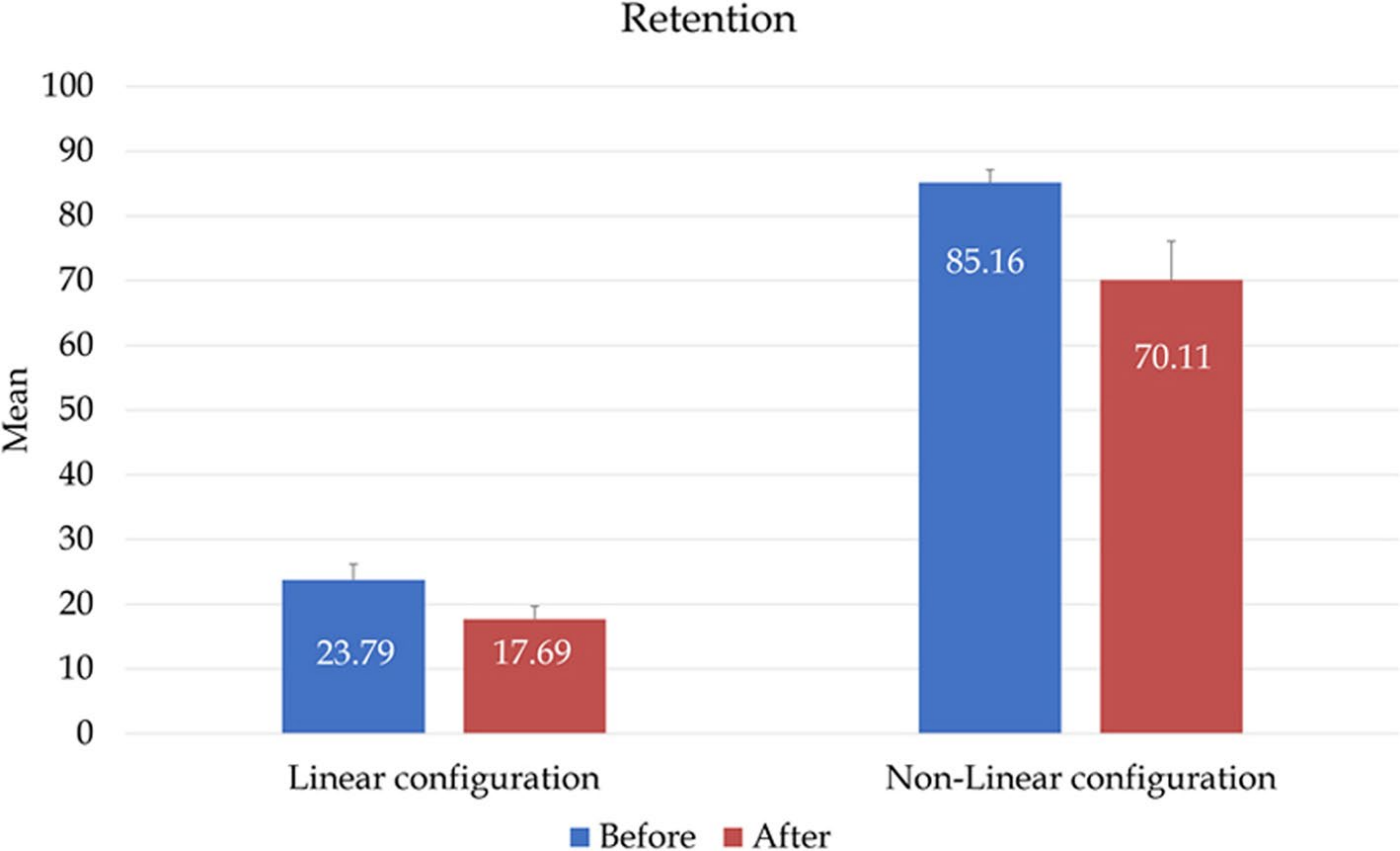
Box plot graph comparing retention of both groups before and after simulation of six months of use

There was a statistically significant difference in the initial retention values between the two groups, as group I (linear configuration) exhibited retentive force values of mean = 23.79 (2.44) N while group II (nonlinear configuration) exhibited more retentive force values of mean = 85.16 (1.94) N with a *p*-value of < 0.0001*. Also, after a cyclic tension-compression test of 500 cycles, each group had a statistically significant difference between initial and final retention, with a *p*-value of -24.87 (10.16) (*p* < 0.009) for group I and 17.49 (7.78) (*p* < 0.009) for group II, indicating a reduction in retention after the tension-compression test. Additionally, a statistically significant difference in final retention was found between the two groups, as group II (nonlinear configuration) showed a higher final retentive force value of mean = 70.11 (5.99) N than group I (linear configuration) of mean = 17.69 (1.98) N with a *p*-value < 0.0001 (Table 1; Fig. 6).

**Table 1:** Comparison of retention between groups

Simulation of 6 months of use	Linear configuration (*n* = 13)		Nonlinear configuration (*n* = 13)		*P* value
	Mean (SD)	Median (IQR)	Mean (SD)	Median (IQR)	
Before	23.79 (2.44)	23.50	85.16 (1.94)	85.10 (3.35)	< 0.0001*
After	17.69 (1.98)	18.03 (3.13)	70.11 (5.99)	69.80 (11.27)	< 0.0001*
*P* value	0.009*		0.009*		
Loss %	-24.87 (10.16)	-28.18 (19.98)	-17.49 (7.78)	-15.29 (14.13)	**0.251**

*Statistically significant differences at *p* value≤0.05

**Fig. 7:**
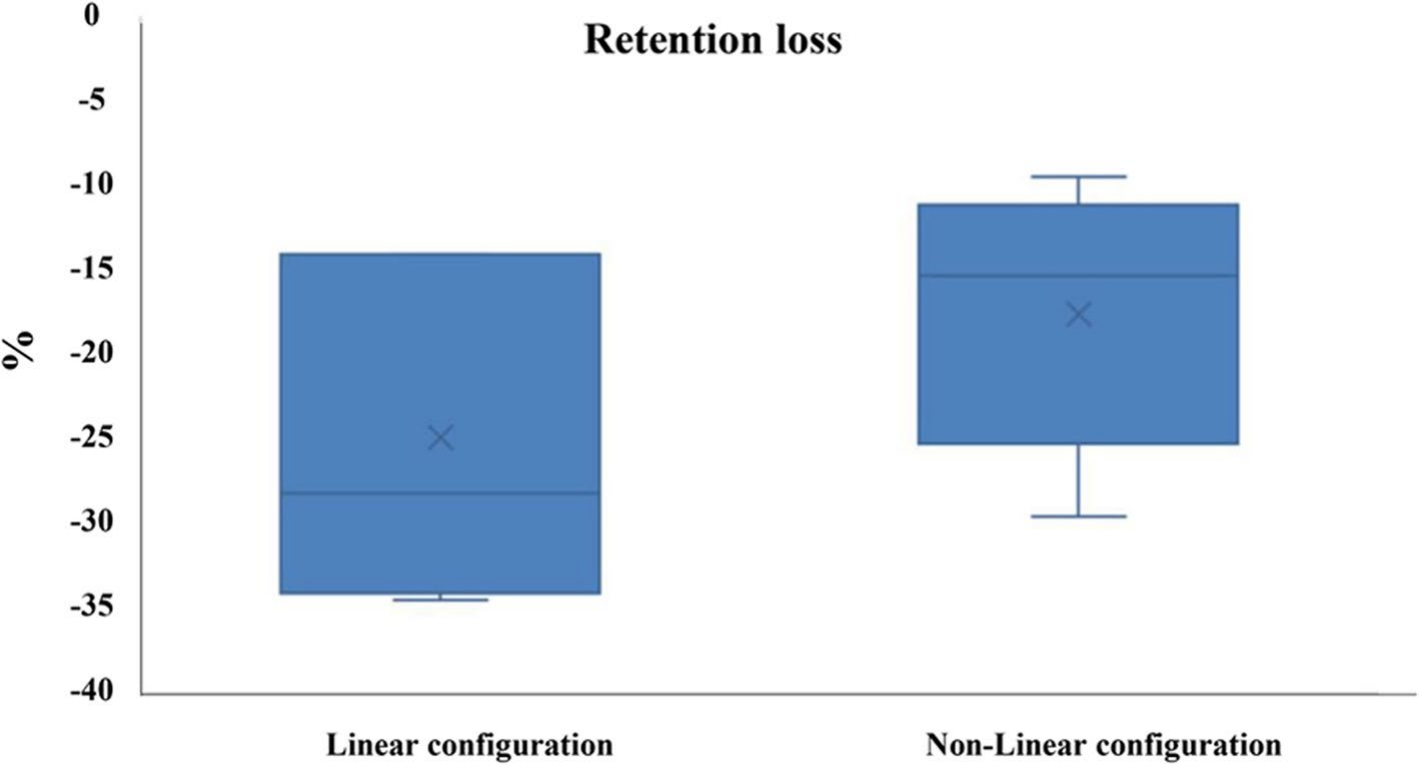
Box plot graph comparing the percentage of retention loss in both groups after simulation of six months of use

These results showed that the nonlinear implant placement configuration for class IIb maxillectomy according to Brown’s classification showed more initial and final retentive force than the linear implant placement configuration (Table 1; Fig. 6). However, after a cyclic tension-compression test of 500 cycles, all implant-retained obturators lost significant portions of their initial retention, but group I (linear configuration) lost a higher percentage of its retention compared to group II (nonlinear configuration) (Table 1; Fig. 7).

The percentage of retention loss at the final stage among both groups showed a non-significant value (*p* = 0.251).

## Discussion

The study showed that there was a statistically significant difference in retention between obturators retained by three implants arranged in linear and nonlinear implant placement configurations therefore the null hypothesis was rejected.

Appropriate and non-invasive treatment options for patients with maxillary defects may include the use of well-designed obturator prostheses for rehabilitation [25].

In the current study, the use of a maxillary arch having maxillectomy class IIb according to Brown’s classification [16], as Sun, Q, et al. showed that class II is the most common maxillectomy defect [26], The epoxy resin model was used for implant placement because it has been reported to have an appropriate elastic modulus (about 20 GPa) for a material similar to bone. Additionally, it was easy to make and durable enough to resist repeated testing [27].

To ensure standardization of all maxillary obturators during construction we duplicated a stone model for each obturator from the epoxy model and during the arrangement of maxillary acrylic teeth, the mandibular standard typodont fully dentate model was kept on the articulator, and all maxillary records bases were changed on the same mounting to preserve the same maxillo-mandibular relationship to unify the occlusion relation for all obturators.

It was reported that the remaining alveolar crest after maxillectomy is suitable for implant placement to increase the comfort of the obturator [28]. Regarding biomechanics, the posterior portions of an edentulous maxilla where the main functional occlusal stress occurs during chewing are ideal for endosseous implants [29]. According to general guidelines for implant insertion, the canine, and first molar are key positions, and implants must be inserted in these sites [30] and it is suggested that the incisor region of the maxilla receive one or two implants to lessen the impact of the arc form [31]. Also, it is reported that because the anterior maxillary segment lies opposite the most retentive section of the defect, which is found along the posterior lateral wall, the residual pre-maxillary segment continues to be the most appropriate position for implants for the majority of maxillectomy patients. Moreover, most patients have a sufficient volume and density of bone in the pre-maxilla, so every attempt is made to maintain this portion of bone as much as possible [11]. Those previous findings guided our study to insert implants at the canine, premolar, and molar in group I and at incisor, premolar, and molar in group II.

An acrylic drilling template was used because only the precise location of the implants was required and a dental surveyor was used to ensure parallelism However, other studies used a surgical guide to provide precise location and angulation of implants [21, 32].

Each implant had a diameter of 3.5 mm and a length of 10 mm. The 10 mm length was chosen since it’s regarded as sufficient for getting an optimal stress distribution around the implants. According to Georgiopoulos et al. [33] reduced bone tissue strain was seen during both immediate and delayed implant loading when implant length increased from 10 mm to 14 mm. However, implants smaller than 10 mm had little effect on the strain field. It was also reported that the buccal and lingual walls at the crest of the intended implant should have at least 1 mm of bone to provide adequate bone thickness and blood supply around the implant for predictable survival [34]. For this reason, a 3.5-mm-diameter implant was used.

The model was fixed to the lower component of the universal testing machine to measure the obturator’s retention and provide a tensile dislodging force to the obturator. The obturator was attached using auto-polymerizing acrylic resin to a T-shaped metal rod that was fixed to the upper component of the UTM. This allowed the tensile force to be applied as much as possible perpendicularly to the occlusal plane to mimic axially directed dislodging forces when an obturator is operating.

A directional dislodgment test was employed in multiple investigations [22, 35–40] to ascertain the prosthesis’s stability and retention.

To replicate the speed at which a prosthesis would be removed from the residual alveolar ridge, the crosshead speed of the UTM was adjusted to 50 mm/min [38]. The peak load to dislodgement was measured on the computer connected to the universal testing machine prior to and following a cyclic tension-compression test in order to determine the beginning and final retentive forces of each implant placement configuration.

The cyclic loading machine replicated the insertion and removal of each obturator during the vertical application of the cyclic tension-compression test. Based on three removal-insertion cycles, an average of 500 cycles were applied to each obturator, representing the number of insertion and removal cycles in 6 months [23, 24].

In the current study, the nonlinear implant placement configuration for class IIb maxillectomy according to Brown’s classification showed more initial and final retentive force than the linear implant placement configuration. Although, after a cyclic tension-compression test of 500 cycles, all implant-retained obturators lost significant portions of their initial retention, group I (linear configuration) lost a higher percentage of their retention than group II (nonlinear configuration). The study’s findings were consistent with biomechanical Class I lever movements [41]. The distance to the anterior tooth serves as the other lever arm, while the posterior extension serves as the first. Rotation can be produced anteriorly or posteriorly, depending on the force exerted on the lever arms. Even with little loading, there will be more denture rotation or dislodgment if the implant (furculum) is positioned far from the loading point (posterior area during mastication).

In group II (nonlinear configuration) increasing the length of the resistance arm by moving the anterior implant more anteriorly results in a greater increase in obturator resistance to dislodgment, as more power would be required to remove the obturator this finding is in agreement with the finding of Alshenaiber, Rafif, Craig Barclay, and Nick Silikas [42].

Numerous investigations [15, 41–43] revealed that the location of the implant has a major impact on the stability and retention of the implant-retained overdenture, and the dislodgment forces increased with an increase in the inter-implant distance. Furthermore, a wide distribution of implants by increasing the distance between the anterior and posterior implants improved the overdenture resistance to anterior-posterior dislodgments, Sadig, Walid [43]. reported that the anterior implant’s presence increased stability by indirectly retaining the overdenture during its posterior dislodgment and those findings supported our results of increased retention among the group of nonlinear implant distribution.

Various research studies [44–46] concluded that metallic components do experience retention loss after wear simulation. Moreover, it was claimed that the wear pattern of these attachments was modulated by the physical characteristics of the attachment alloys, specifically the modulus of elasticity. Those findings are consistent with the current study, which found that the retention force decreased with time in both implant placement configurations. Cyclic loading on the nylon components causes surface alterations that can also be used to explain this retention loss [46, 47]. But despite that, the nonlinear implant configuration showed less loss of retention, which supports the advice to use a nonlinear configuration for implant-retained obturators for completely edentulous unilateral maxillary defect (Brown’s class IIb).

The current study showed some limitations in that the magnitude or frequency of the para-axial dislodgment forces cannot be measured in a laboratory setting. Since these forces are known to arise during function, it is recommended to be measured by other studies because multiple strategies were tried to improve the stability of the removable prosthesis by resisting these unfavorable forces.

It is recommended to perform a clinical long-term in vivo study for nonlinear implant placement configuration for class IIb browns classification. It is also recommended to test the effect of the implant number and angulation on the retention of the obturators.

## Conclusion

Based on the findings of this invitro study, for obturators with class IIb maxillectomy according to Brown’s classification, it can be concluded that:

it was determined that the nonlinear implant placement configuration had a superior initial retention force and less retention force loss after usage than the linear implant placement configuration. Therefore, the authors suggest using that nonlinear implant placement configuration for achieving favorable retention for implant-retained obturators for completely edentulous unilateral maxillary defects (Brown’s class IIb).

## Data Availability

The datasets used and/or analyzed during the current study are available from the corresponding author upon reasonable request.

## Data availability

Data is provided within the manuscript or supplementary information during the current study are available from the corresponding author on reasonable request.
